# Differential Expression of MUC12, MUC16, and MUC20 in Patients with Active and Remission Ulcerative Colitis 

**DOI:** 10.1155/2015/659018

**Published:** 2015-12-06

**Authors:** Jesús K. Yamamoto-Furusho, Ilse Ascaño-Gutiérrez, Janette Furuzawa-Carballeda, Gabriela Fonseca-Camarillo

**Affiliations:** ^1^Inflammatory Bowel Disease Clinic, Department of Gastroenterology, Instituto Nacional de Ciencias Médicas y Nutrición Salvador Zubirán, 14080 Mexico City, Mexico; ^2^Department of Immunology and Rheumatology, Instituto Nacional de Ciencias Médicas y Nutrición Salvador Zubirán, 14080 Mexico City, Mexico

## Abstract

*Background*. Patients with UC have shown an important defect in the secretion and maintenance of the mucosal barrier as part of inadequate expression of mucin genes. The aim of the present study was to determine the expression of MUC12, MUC16, and MUC20 in colonic tissue from patients with UC in regard to their clinical outcomes.* Methods*. We included a total of 40 patients with UC and 30 normal controls. Mucin gene expression was performed by RT-PCR and protein expression was detected by immunohistochemistry.* Results*. Patients with active UC showed no significant expression of MUC12 gene in mucosa compared to the group of patients with UC in remission and the normal control group. MUC16 gene expression was significantly increased in the UC active and remission groups compared to the normal control group (*P* = 0.03). MUC20 gene expression was found significantly decreased in patients with active UC compared to both remission group (*P* = 0.001) and normal controls (*P* = 0.001). Furthermore, an association was found between MUC20 gene expression and the presence of histological remission in patients with UC (*P* = 0.003, OR = 0.37).* Conclusions*. An increased gene expression of MUC16 and MUC20 was found in patients with remission UC.

## 1. Introduction

Inflammatory bowel disease (IBD) consists of ulcerative colitis (UC) and Crohn's Disease (CD), progressive and inflammatory disorders of the intestinal tract. Ulcerative colitis (UC) is limited to the colon and involves diffuse mucosal inflammation and its etiology remains unknown [1]. The UC pathogenesis involves an inappropriate immunological response (antigen presentation and tolerance) and an imbalance of mucin expression. Mucins (MUC) are high-molecular-weight glycoproteins and constitute the first line of innate immunity. So far, there are at least 21 human MUC known [2]. Mucins are produced by multiple cells including goblet cells, epithelial cells, enterocytes, and resting or activated mononuclear cells in the intestinal tract. Mucin functional diversity is related to its structure. There are 2 subtypes: secretory and membrane-bound which constitute the main component of the mucous layer lining the GI tract. The secretory mucins forming the mucus barrier include MUC2, MUC5AC, MUC5B, MUC6, and MUC19 and constitute large polymers rich of proteoglycans that are highly hygroscopic and hydrophilic. Membrane-bound mucins (MUC1, MUC3, MUC4, MUC12, MUC13, MUC15, MUC16, MUC17, and MUC20) are anchored to the apical membrane of epithelial cells and form the glycocalyx. Nonetheless, they are poorly characterized [3].

Secretory membrane-bound mucins in the normal gut are involved in cell signaling because they interact with membrane receptors and have several functions such as adhesion, growth, and immune modulation [4]. Their expression and secretion are constitutive and/or regulated by pathogens, IL-1*β*, TNF-*α*, and Th2 cytokines [5].

Altering of mucus barrier functions, composition, and lubrication, depriving the epithelial gut, and favoring the contact with toxins, cytokines, and commensal or pathogenic microorganisms through adhesins, which induces acute and chronic inflammation in the intestine. Patients with UC have shown an aberrant MUC synthesis and degradation as well as alterations of mucin's glycoprotein structure (o-glycosylation, sulfation, and silylation) leading to a deficient mucous barrier [1, 2]. Some groups have described a reduction in MUC1, MUC2, and MUC3 and MUC17 gene expression in both inflamed and nonaffected mucosae from UC and CD patients [2, 6–8]. MUC2 is the predominant sulfated component of mucin layer in healthy colon. In active UC, MUC2 production and secretion by goblet cells are reduced, and this induces synthesis of MUC5AC and MUC6 [6]. On the other hand, it has been reported that MUC1 is overexpressed in severe UC. This finding correlates with an elegant experiment demonstrating that MUC1^−/−^ mice model is less susceptible to dextran sulfate sodium induction of colitis and that fewer activated T cells were recruited to the inflamed mucosa [5]. MUC17 in healthy intestine is one of the major membrane-bound mucins. However, its expression is reduced in UC patients [6]. MUC13 was increased in active UC but MUC3 did not change. The MUC4 was similarly increased, especially when associated with the development of neoplasia. An aberrant expression of MUC5AC was found in UC, as well as in CD [9, 10]. Previous studies from Moehle et al. described significant downregulation of MUC12 and MUC20 in colon and ileum in patients with Crohn's Disease [6].

MUC12 is a protein-coding gene, whose function is involved in the epithelial cell protection [9]. The function of MUC16 is thought to provide a protective, lubricating barrier against particles and infectious agents at mucosal surfaces [10].

MUC20 is a protein-coding gene that contains at least four exons and is located close to MUC4 on chromosome 3q29. It has been reported that MUC20 is oversynthesized in gastric, ovarian, endometrial, and colorectal cancer and predicts recurrence and poor outcome [11].

Little is known about the MUC12, MUC16, and MUC20 expression and their potential role in the physiopathology of UC. Thus, the aim of the present study was to characterize the changes in gene and protein expression of these mucins in colonic tissue from patients with UC in regard to their clinical outcomes.

## 2. Materials and Methods

### 2.1. Study Subjects

We studied 20 patients with active UC, 20 patients in remission UC, and 30 normal controls without inflammation. All individuals belonged to the Inflammatory Bowel Disease Clinic at the Instituto Nacional de Ciencias Médicas y Nutrición Hospital. The diagnosis of UC was done by the presence of the following criteria: macroscopic appearance by endoscopy and biopsy compatible with UC and history of diarrhea or blood in stool. Demographic and clinical variables were collected from personal interview and clinical records. The clinical and endoscopic activity was evaluated by Mayo [12] and histological activity by Riley score.

The tissue control group consisted of noninflamed controls (no documented inflammatory disease) undergoing colonoscopy for evaluation of anemia and screening of polyps. All individuals were included as controls if no pathological findings were found.

### 2.2. Sample Processing and Gene Expression Analysis

The colonic biopsies for gene expression analysis were taken with colonoscopy and were immediately placed in preserver of RNA (Ambion, Austin, TX, USA) and stored at −70°C until processing. Then, total RNA was isolated using High Pure RNA Tissue (Roche Diagnostics, Mannheim, Germany). Colonic biopsies samples were disrupted in 400 *μ*L Lysis Buffer and homogenized. The RNA was isolated by binding to the glass fibers prepacked in the High Pure Filter Tube. Residual contaminating DNA was digested with DNase I. Bound RNA was washed, thereby purified from salts, proteins, and other cellular impurities. Finally, we added 100 *μ*L of Elution Buffer for the elution of RNA.

Two hundred nanograms of total RNA was reverse-transcribed into cDNA with random hexamer primers (Roche Diagnostics, Mannheim, Germany).

The MUC gene relative expression was measured by quantitative real-time polymerase chain reaction (RT-PCR). Reference genes RPLP0, ACTB, and GAPDH transcripts were used for relative quantification and quality controls. For qPCR assays quality control, determination of linearity and reproducibility was evaluated (VC < 10%). The mRNA relative quantification of target genes was conducted using the LightCycler software 4.1, according to the 2-delta-delta Ct method.

We used the following primers: MUC12 forward: cctggaaaccttagcaccag and reverse: gacagacgcattgttttccat, MUC16 forward: tggggaccaccaattctatg and reverse: atggctgggagtggattg, MUC20 forward: tatgtgccgtggaggattc and reverse: ttgctgtacgtgtctaacttcaatc, IL-6 forward: tctgctcccacaatgaaacat and reverse: gaaggcagcaggcaacac, IL-8 forward: agacagcagagcacacaagc and reverse: atggttccttccggtggt, GADPH forward: gcccaatacgaccaaatcc and reverse: agccacatcgctcagaca, RPLP0 forward: gaagctctatctcgcctcca and reverse: agcaggcaacaccaggag, and ACTB forward: caaccgcgagaagatgac and reverse: gtccatcacgatgccagt, for normalization as also shown in Table 1.

The PCR amplification of mucin genes was carried out with 20 ng of cDNA, 200 nM forward and reverse primer, and Taqman Master Mix (Roche Diagnostics, Mannheim, Germany) in a final volume of 10 *μ*L. PCR reactions were run in a Light Cycler 480 (Roche Diagnostics, Mannheim, Germany) for 45 cycles; each cycle consists in denaturation for 15 seconds at 95°, primer annealing for 15 seconds at 55°, and extension for 30 seconds at 72°C and cooling for 30 seconds at 40°C.

### 2.3. Immunohistochemistry

All tissues obtained were formalin fixed and paraffin embedded. Five *μ*m tissue sections were rehydrated using xylene and graded ethanol series. After deparaffinizing and demasking of antigens with 10 mM citrate buffer, pH 6.0, the slides were then soaked in 1% hydrogen peroxide in methanol for 20 min at room temperature to block endogenous peroxidases and then washed with PBS. Slides were blocked with 10% normal donkey serum and were incubated with a primary mouse monoclonal anti-human MUC12 mAb (Santa Cruz Biotechnology, Santa Cruz, CA), MUC16 mAb (ABCAM), and MUC20 mAb (ABCAM), overnight at 4°C, and then samples were incubated with the secondary goat anti-mouse IgG (Santa Cruz Biotechnology) diluted 1 : 2000 for 1 hour at room temperature. The slides were treated consecutively with streptavidin-horse-radish peroxidase conjugate diluted 1 : 1000 for 45 minutes at room temperature. Next, tissues were incubated using 6 mg 3,3′-diaminobenzidine (DAB) substrate and H_2_O_2_; the color reaction was allowed to develop for 5–10 min. After the staining reaction, the slides were washed thoroughly in tap water, counterstained with Mayer's hematoxylin for 2 minutes and saturated lithium carbonate solution for 10 seconds, and mounted with cover slides. Substitution of primary antibodies with PBS was used as the reactive blank. Negative control staining was performed with normal human serum diluted 1 : 100, instead of primary antibody. Both controls excluded nonspecific staining or endogenous enzymatic activities. Scoring of immune-stained sections was done by conventional light microscopy in a blinded manner. MUC12-, MUC16-, and MUC20-producing cells were counted in at least three optical fields from each slide in ×320 high-power magnifications. The average values per slide were used for statistical analysis. Results are expressed as the mean ± standard error of the mean (SEM) of cells quantified by the program Image Pro-Plus version 5.1.1.

### 2.4. Ethical Considerations

The protocol was approved by the ethical medical committee in our institution and it was according to the principles expressed in the Declaration of Helsinki, 1989. Only patients who gave a written informed consent were recruited for this study.

### 2.5. Statistical Analysis

Descriptive statistics were performed, and categorical variables were compared using the *χ* ^2^ test or Fisher's exact test. ANOVA on ranks by Holm-Sidak Method and Dunn's test was performed for all pairwise multiple comparison procedures. Statistical analysis was done using the Sigma Stat 11.2 program (Aspire Software International, Leesburg, VA, USA). Data were expressed as the median, range, and mean ± standard deviation (SD)/standard error of the mean (SEM). *P* ≤ 0.05 was considered as significant.

## 3. Results

### 3.1. Demographic and Clinical Characteristics

A total of 40 patients with UC (19 men and 21 women with a mean age of 42 years) and 30 normal controls (16 men and 14 women with a mean age of 53 years) were evaluated. Regarding the grade of disease activity, 20 had active disease and 20 were in remission according to Mayo score. The extent of disease was evaluated by using total colonoscopy and biopsies were taken from different segments of colon in all cases. The Montreal classification was used to define the extent of UC: 45% had pancolitis (E3); 20% had left-sided colitis (E2); and 35% had proctitis (E1).

Histological analysis of patients with active UC showed 20% with mild activity, 40% with moderate activity, and 40% with severe histological inflammation. On the other hand, the histological analysis of UC patients in remission shows an association with the gene expression of MUC16 (*P* = 0.0003, OR = 0.37). As part of the clinical features, 21 of our UC patients (52%) had extraintestinal manifestations.

Regarding the medical treatment, all patients were taking sulfasalazine or 5-aminosalicylic acid (5-ASA); 47.5% used oral or systemic steroids; 30% were taking azathioprine; and 2.5% had anti-TNF therapy.

The demographic and clinical characteristics from all UC patients are shown in Table 2.

### 3.2. Mucin 12, Mucin 16, and Mucin 20 Gene and Protein Expression in Colonic Tissue of UC Patients

**Figure 1:**
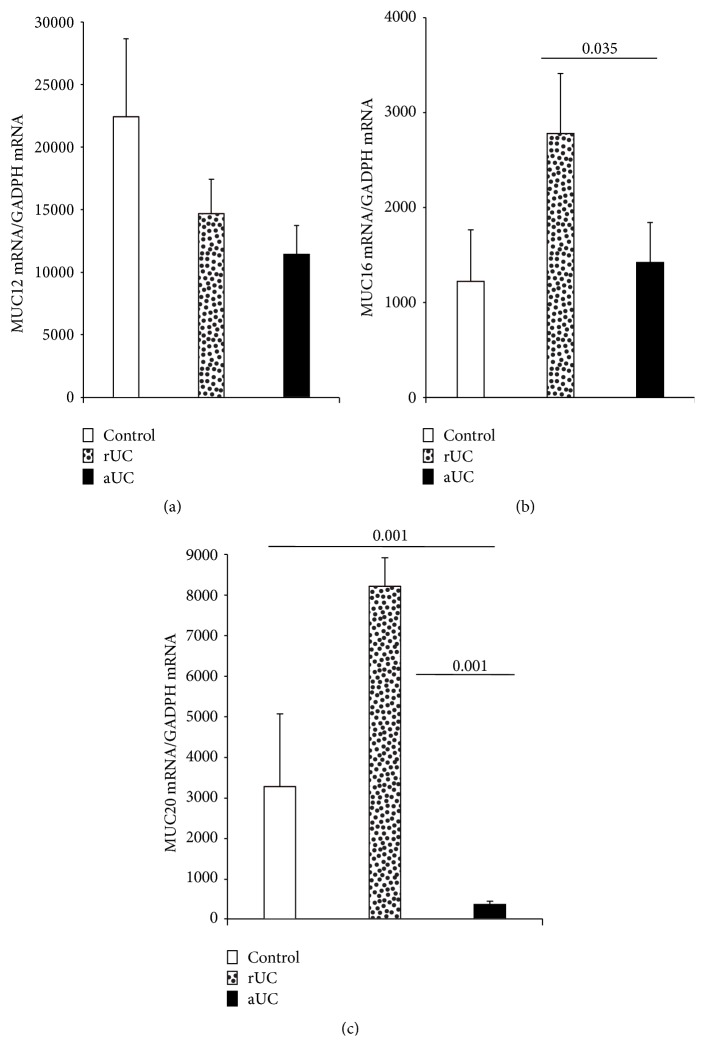
(a) MUC12, (b) MUC16, and (c) MUC20 mRNA levels in rectal mucosa from patients with ulcerative colitis (*N* = 40) and controls (*N* = 30). RT-qPCR was performed to assess mRNA levels in colonic mucosa biopsies from UC patients; bars show means with standard error of the mean of MUC20 transcript levels with GAPDH as housekeeping gene determined by 2ΔΔCt.

**Figure 2:**
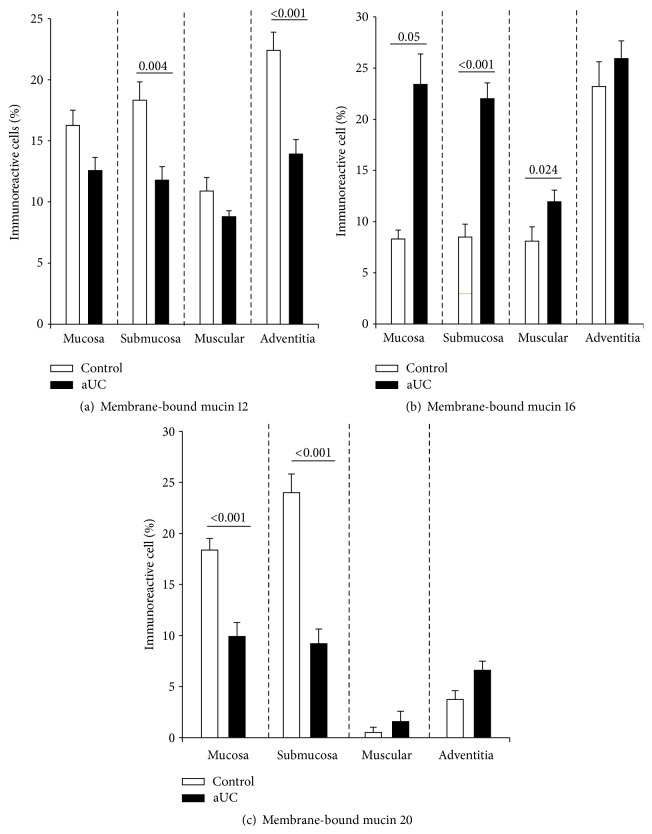
(a) Percentage of MUC12, (b) MUC16, and (c) MUC20—expressing cells in active UC patients (*N* = 10). Results are expressed as median (black line).

**Figure 3:**
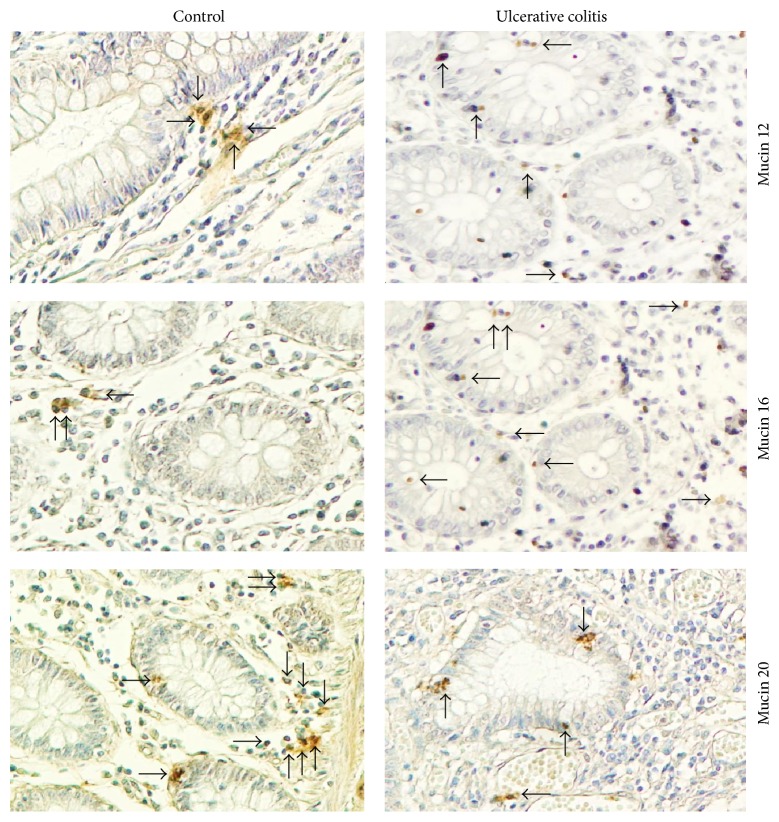
MUC12, MUC16, and MUC20—expressing cells in colonic tissue from patients with ulcerative colitis. Representative immunoperoxidase analysis in noninflamed colonic tissue (*N* = 10) (left panel) and active ulcerative colitis (*N* = 10) (right panel). Arrows depict immunoreactive cells in mucosa, submucosa, muscular, and adventitia layers. Original magnification was ×320.

Patients with active UC showed no significant expression of MUC12 gene in mucosa compared to the group of patients with UC in remission and the normal control group (Figure 1(a)). No association was found between MUC12 gene expression and the clinical features of UC. In the immunohistochemistry, colonic tissue of UC patients had abundant inflammatory infiltrates, predominantly mononuclear cells, which extended from the serosa to mucosa, being more abundant in the epithelium and in the serosa. In all cases, a significant destruction of the mucosa with decreased production of MUC12 protein in epithelial cells and mononuclear infiltrate in submucosa (*P* = 0.004) and adventitia (<0.001) of colon specimen of active UC (Figures 2(a) and 3) was shown.

MUC16 gene expression was significantly increased in the UC remission group compared to the normal control group and active UC group (*P* = 0.03) as shown in Figure 1(b). No association was found between MUC16 gene expression and the clinical features of UC. In order to evaluate the gene expression findings of differential mucin regulation in the intestine, MUC16 was identified by immunohistochemistry. The protein expression of MUC16 was higher in epithelial cells and mononuclear infiltrate from mucosa (*P* = 0.05), submucosa (*P* < 0.001), and muscular layer (*P* = 0.024) in colonic specimen from active UC patients compared to the control group (Figures 2(b) and 3).

Finally, MUC20 gene expression was found significantly increased in patients with remission UC compared to active UC (*P* = 0.001) and normal controls (*P* = 0.001). Furthermore, an association was found between MUC20 gene expression and the presence of histological remission in patients with UC (*P* = 0.003, OR = 0.37) as shown in Figure 1(c). In this instance, the protein expression of MUC20 showed a decreased production in epithelial and mononuclear cells from mucosa (*P* < 0.001) and submucosa (*P* < 0.001) in patients with active UC compared to the control group (Figures 2(c) and 3).

### 3.3. IL-6 and IL-8 as Intestinal Inflammatory Markers in UC Patients

**Figure 4:**
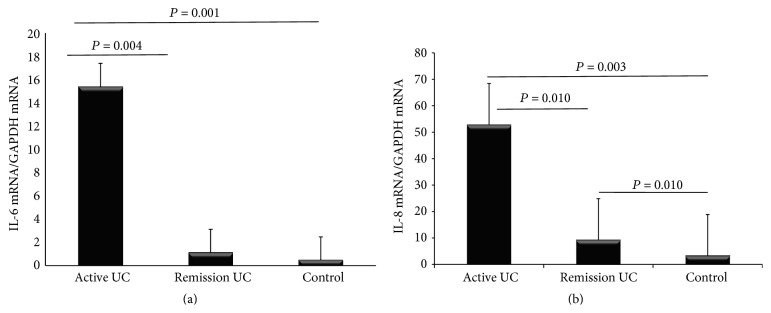
IL-6 and IL-8 mRNA levels in colonic mucosa from patients with ulcerative colitis and controls. RT-qPCR was performed to assess mRNA levels in colonic mucosa biopsies from UC patients, bars show means with standard error of the mean of IL-6 and IL-8 transcript levels with GAPDH, RPLP0, and ACTB as housekeeping gene determined by 2ΔΔCt, and differences among groups were assessed by Kruskal-Wallis test.

To further evaluate the relation of mucins mRNA levels in UC and intestinal inflammation in UC patients, we also measure IL6 and IL-8 mRNA levels. The IL6 mRNA levels showed an important capacity to differentiate between remission and active disease. The IL6 and IL-8 mRNA levels were higher in the active group compared to remission of UC, Figures 4(a) and 4(b).

## 4. Discussion

The findings of this study showed a significant increase of the production of mucins 16 and 20 mainly produced by epithelial cells and mononuclear infiltrate. No increase was found in the production of mucin 12. Interestingly, the production of mucin 20 was found to be associated with the presence of histological remission in patients with UC.

In normal conditions, the gastrointestinal tract is protected by a mucus barrier with both secreted and cell-surface mucins contributing to the exclusion of luminal microbes and toxins. Alterations in the structure and/or quantity of mucins modify the barrier function of mucus and could play a role in initiating and maintaining mucosal inflammation in IBD [13, 14]. In the IBD a loss of tight junctions has been lead an increase in the intestinal permeability, it could be explained by a loss of cell polarity and hence overexpression of cell surface mucins [6, 14].

Patients with UC showed a significant decrease in the gene and protein expression and production of mucin 20 in epithelial cells from mucosa as well as the submucosa in patients with active disease compared to both in remission group and in normal controls. This gene was associated with histological remission in patients with UC where expression of MUC20 might have a protective role in patients with UC. This study is the first depiction of the association of high mucin 20 expression associated with histological remission in UC patients. On the other hand, the overexpression of MUC20 was associated with recurrence and poor outcome in patients with colorectal cancer [15].

In contrast, MUC16 gene and protein expression were significantly increased in active UC compared to the control group at different levels such as mucosa and submucosa. Nevertheless, no association was found between MUC16 gene expression and clinical characteristics of UC. MUC16 is synthesized in several layers of the colon such as mucosa, submucosa, and muscular in active UC patients. In contrast, a previous study demonstrated that overexpression of MUC16 was associated with lower survival rate in patients with endocervical adenocarcinoma [16].

MUC12 gene and protein expression were decreased in submucosa and adventitia of colonic tissue from active UC patients as compared to normal controls. However, no association was found between decreased MUC12 gene expression and clinical features of UC. The production of MUC12 is mainly by epithelial cells from ocular and respiratory tract and no production of MUC12 in goblet cells [17].

Finally, it is important to note that this study provides evidence about the production of MUC20, MUC16, and MUC12 from enterocytes and confirms that the MUC12 and MUC20 are produced by epithelial cells from colon and rectal areas compared to goblet cells that secretes MUC1, MUC2, MUC3, and MUC5AC [3, 18].

Previously it has been reported that low expression of MUC9 gene in UC patients (active and remission) compared to control individuals suggests that MUC9 has an aberrant expression contributing to a defect in the mucus layer and altered physical barrier between luminal contents and the mucosal surface. According to other studies in pediatric and adult patients with IBD, mucins have been involved in the development of IBD such as MUC1, MUC2, MUC5AC, and MUC12 [14, 19–21].

Since this is an observational cross section study, it should furthermore be stated that long-term follow-up studies are needed in order to make prognostic predictions in a given patient population.

In conclusion, MUC16 and 20 showed an increased expression in the colonic mucosa from patients with remission UC. This might be explained as a protection mechanism in patients with quiescent UC.

## Figures and Tables

**Table 1:** Primers designs from Universal ProbeLibrary.

Gene	GenBank	Oligo left	Oligo right	UPL	Amplicon size
MUC12	NM_001164462.1	cctggaaaccttagcaccag	gacagacgcattgttttccat	72	cctggaaaccttagcaccagggttgtgccaggaaggacaaatttggaatggaaaacaatgcgtctgtc

MUC16	NM_024690.2	agtggaccttgggacctca	gagagggccagcagatgtag	58	agtggaccttgggacctcagggactccatcctccctccccagccctacatctgctggccctctc

MUC20	NM_001098516.1	tatgtgccgtggaggattc	ttgctgtacgtgtctaacttcaatc	7	tatgtgccgtggaggattcaaatctgtctcttctcccagggattgaagttagacacgtacagcaa

IL-6	NM_000600.3	gatgagtacaaaagtcctgatcca	ctgcagccactggttctgt	68	gatgagtacaaaagtcctgatccagttcctgcagaaaaaggcaaagaatctagatgcaataaccacccctgacccaaccacaaatgccagcctgctgacgaagctgcaggcacagaaccagtggctgcag

IL-8	NM_000584.2	agacagcagagcacacaagc	atggttccttccggtggt	72	agacagcagagcacacaagcttctaggacaagagccaggaagaaaccaccggaaggaaccat

GAPDH	NM_002046.3	agccacatcgctcagaca	gcccaatacgaccaaatcc	60	agccacatcgctcagacaccatggggaaggtgaaggtcggagtcaacggatttggtcgtattgggc

RPLP0	NM_053275.3, NM_001002.3	agcaggcaacaccaggag	gaagctctatctcgcctcca	6	tctacaaccctgaagtgcttgatatcacagaggaaactctgcattctcgcttcctggagggtgtccgcaatgttgccagtgtctgtctgcagattg

ACTB	NM_001101.3	gtccatcacgatgccagt	caaccgcgagaagatgac	64	ccaaccgcgagaagatgacccagatcatgtttgagaccttcaacaccccagccatgtacgttgctatccaggctgtgctatccctgtacgcctctgg

**Table 2:** Clinical and demographic characteristics of ulcerative colitis patients.

Characteristics	Number of patients
Gender (M/F)	19/21
Current age (mean years; range)	40 (18–65)
Age at diagnosis (mean years; range)	24 (18–55)
Disease duration	4 ± 2 years
Disease activity (active/remission)	20/20
Disease extension: distal colitis/pancolitis	22/18
Endoscopic activity (inactive/mild/moderate/severe)	20/4/8/8
Histological activity (inactive/mild/moderate/severe)	20/4/8/8
Current therapy	
*5*-Aminosalicylate	40
Corticosteroids	12
Azathioprine	15
Extraintestinal manifestations (absent/present)	19/21
Control group	
Number of patients, sex (M/F)	9/21
Mean age (years; range)	53 (19–73)

## Abbreviations

IBD: Inflammatory bowel disease
UC: Ulcerative colitis
RT-PCR: Real-time polymerase chain reaction
MUC: Mucin.
